# Relationship between Height Preferences and Endorsement of Gender Norms

**DOI:** 10.1007/s12110-025-09504-x

**Published:** 2025-10-07

**Authors:** Alexandra Dial, Gillian R. Brown

**Affiliations:** School of Psychology & Neuroscience, University of St Andrews, South Street, St Andrews, Fife, KY16 9JP UK

**Keywords:** Attractiveness, Mate choice, Cultural evolution, Feminism, Social norms, Height

## Abstract

Height preferences when choosing a partner might reflect adaptive mating strategies, whereby tall men are deemed attractive to potential partners due to links with health and resource acquisition. However, height preferences are highly variable across populations and could reflect socially constructed gender norms. We examined the relationship between ideal partner height, the importance placed on partner height and endorsement of traditional gender norms. Participants (*n* = 242; 18-39yrs; UK-based, heterosexual) completed (i) five height-related questions (including own height, ideal partner height, maximum/minimum acceptable height), (ii) three gender norm questionnaires (sexist attitudes, feminist attitudes and alignment with masculine/feminine gender roles), and (iii) two open-ended questions about why height is important. Although ideal height ratio did not correlate with any gender role endorsement measures in either women or men, women who placed greater importance on height scored higher on sexism, lower on feminism and were less likely to find a short partner acceptable than women who placed less importance on partner height. Men who placed greater importance on height, and men who described themselves as more traditionally masculine, were less willing to accept a tall partner than men who scored lower on these measures. Women who rated height as important wanted to feel ‘feminine/protected’, whereas men wanted to feel ‘masculine/dominant’. In this study, the ‘male-taller’ preference was exhibited, with women’s preferences for tall partners being stronger than men’s preferences for short partners. Height preferences were related to gender norm endorsement, suggesting that gene–culture co-evolutionary processes could potentially influence human height dimorphism.

## Introduction

Across mammals, males tend to be larger than females (Slavenko et al., 2024), and this size dimorphism has been attributed to sexual selection in most mammalian species (Winkler et al., 2024). For instance, in primates, male body size positively correlates with levels of agonistic male-male competition for mating opportunities (Plavcan, 2001). In human beings, men are taller, on average, than women (Stulp & Barrett, 2016). However, compared to other primate species and extinct hominins, size dimorphism in human beings is relatively modest (Plavcan, 2012; Ruff & Wood, 2023; except for some measures of strength/muscularity: reviewed by Lidborg et al., 2022). Based on cross-cultural datasets, the average ratio of male to female height is around 1.07 (Gustafsson & Lindenfors, 2004), which equates to women reaching around 93% of average male height. In humans, male tallness might serve as an indicator of health and access to resources, given that human height is related to early-life nutrition, health markers and socioeconomic status in both men and women (Perkins et al., 2016; Thompson et al., 2023). Thus, women might gain fitness benefits from preferring tall, healthy partners who can provide access to resources. Taller men are sometimes, but not always, reported to have higher reproductive success than shorter men (e.g., Song et al., 2021; reviewed by Lidborg et al., 2022), whereas height in women does not appear to be consistently associated with offspring numbers (e.g., Murasko, 2022; Sear, 2010; Stulp et al., 2012).

Several lines of evidence indicate that height is an important characteristic that people consider when choosing a potential romantic partner (Lidborg et al., 2022; Stulp & Barrett, 2016). For instance, in questionnaire studies of Western samples, women often state that they want a partner taller than themselves, whereas men state a preference for a shorter partner (e.g., Higgins et al., 2002; Salska et al., 2008; Shepperd & Strathman, 1989; Swami et al., 2008). In online dating profiles and personal advertisements, women tend to seek tall men, and men seek short women (e.g., Kirk & Gupta, 2022; McIntosh et al., 2011; Oda, 2001 ), and taller men and shorter women receive more positive responses to their personal ads and have higher dating success (e.g., Lynn & Shurgot, 1984; Pawlowski & Koziel, 2002; Rhodes et al., 2005; Shepperd & Strathman, 1989). When presented with line drawings of human figures, attractiveness ratings are highest when the male figure is taller than the female figure (e.g., Fink et al., 2007; Varella Valentova et al., 2016), which suggests that this combination of heights is deemed most appropriate for heterosexual couples. This expectation has been referred to as the ‘male-taller’ norm (Gillis & Avis, 1980). This social norm appears to be reflected in real-life choices, given that ‘female-taller’ partnerships are significantly less common in Western populations than would be expected by chance (e.g., USA: Gillis & Avis, 1980; UK: Sear et al., 2004; Stulp et al., 2013a).

However, height preferences are not consistent across all human populations. In some societies, preferences for a male-taller partnership are much weaker than in Western countries (e.g., Sorokowski & Butovskaya, 2012; Sorokowski & Sorokowska, 2012; Sorokowski et al., 2012, 2020). For example, among the Cook Islanders of the South Pacific, around a third of both women and men preferred the set of line drawings of human figures where the partners were the same height, whereas fewer than one in ten Norwegian participants preferred this option (Sorokowski et al., 2020). Similarly, among the Yali tribe of Papua, neither men nor women showed an overall preference for male-taller partnerships when shown sets of human figures (Sorokowski & Sorokowska, 2012). In contrast, male-taller preferences were particularly strong in some small-scale societies; for example, among the Hadza of Tanzania, both men and women preferred a partnership in which the man was represented as being substantially taller than the woman (Sorokowski et al., 2015a). Whether real-world partnerships conform to the male-taller norm also demonstrates variability across societies; for example, in contrast to data from Western populations (e.g., Gillis & Avis, 1980; Sear et al., 2004), the proportion of real-life partnerships in which the woman is taller is the same proportion that would be expected by chance in several small-scale societies (e.g., Becker et al., 2012; Sear & Marlowe, 2009; Sear et al., 2004). Social and cultural factors could play an important role in shaping height preferences.

Within populations, the extent to which individuals endorse social norms is likely to influence their partner preferences. Previous research has reported that women and men who endorse sexist gender norms are more likely to conform to ‘traditional’ partner preferences than are individuals who score low on sexism measures (e.g., Alba et al., 2023; Eastwick et al., 2006; Johannesen-Schmidt & Eagly, 2002; Thomae & Houston, 2016; Travagila et al., 2009). For instance, in women, the extent to which individuals hold sexist attitudes correlated with greater preference for a partner with good financial prospects (Eastwick et al., 2006), while, in men, sexist attitudes were associated with assigning greater importance to the physical attractiveness of potential partners (Johannesen-Schmidt & Eagly, 2002). These findings are consistent with the idea that sexist attitudes align with expectations of ‘traditional’, stereotyped attributes in potential romantic partners. However, the majority of these studies have focused on characteristics such as access to resources and good looks (reviewed by Sibley & Overall, 2011), rather than height, which means that the relationship between the endorsement of gender norms and height preferences is yet to be fully explored. Endorsement of traditional gender norms is predicted to be associated with preference for a larger height disparity within partnerships and/or greater importance being placed on the height of a potential partner.

Only a small number of studies have investigated the link between gender role attitudes and height preferences (Salska et al., 2008; Swami et al., 2008, 2010; Tao, 2020). Swami and colleagues (2008) reported that, in a UK-based sample, both women and men who scored higher on a measure assessing their negative attitudes towards feminine behaviour in men stated a preference for a larger height difference between themselves and their partner. Similarly, ideal difference in height was positively correlated in both genders with their scores on a measure of sexist attitude endorsement (Swami et al., 2010). In contrast, Salska and colleagues (2008) did not find a correlation between ideal height ratio and endorsement of traditional male roles in a US-based sample. Using Taiwanese survey data, Tao (2020) found that men, but not women, who endorse traditional gender norms stated a narrow range of acceptable partner heights. Therefore, although height preferences do appear to be related to endorsement of traditional gender norms, the pattern of findings has differed between studies. In addition, none of these previous studies examined the extent to which participants endorsed feminist attitudes, rather than the extent to which they rejected sexist attitudes. Wareham and colleagues (2024) found that feminist, but not sexist, attitudes correlated with the extent to which men place emphasis on the physical attractiveness and age of potential partners. Thus, the associations between gender role attitudes and height preferences might differ between measures of sexist and feminist attitudes.

Stereotypes about the roles of men and women in romantic partnerships might be reinforced by being in a male-taller partnership. In a qualitative study on US college students, Yancey and Emerson (2016) found that men perceived themselves to be protectors and more masculine when they were taller than their partner, while women reported feeling more secure, protected and feminine when they were shorter than their partner. Similarly, young adults in the Philippines cited protection and power, as well as social acceptability and aesthetics, as reasons why height is important to them in relationships (Taduran, 2021; *n* = 100). These findings suggest that the extent to which individuals personally align with traditional expectations about masculinity and femininity, which might vary across cultures, could be related to their height preferences. In a study in Poland, Sorokowski and colleagues (2015b) reported that women who described themselves as less aggressively dominant tended to prefer taller partners, relative to their own height, than did more dominant women. However, dominance covers only one aspect of gender norms. No studies have tested the prediction that men who broadly describe themselves as ‘masculine’, and women who describe themselves as ‘feminine’, are more likely to exhibit male-taller preferences than those who do not associate themselves with these terms.

This study examined the relationship between height preferences and (a) endorsement of sexist and feminist social norms, and (b) alignment with masculine and feminine gender roles in a UK-based sample of young, heterosexual adults (aged 18–39 years old). Using an online survey, participants were asked to complete (i) a set of five height-related questions (i.e., own height, ideal partner height, importance placed on height, and maximum and minimum acceptable partner height), (ii) questionnaires about their endorsement of sexist and feminist social norms, and the extent to which they would describe themselves as traditionally masculine (male participants) or feminine (female participants); and (iii) two free-response questions that asked why height was important to them when selecting someone to date and whether they considered that social norms influence their preferences. We predicted that, on average, (i) women would prefer a partner taller than themselves, while men would prefer a partner shorter than themselves (i.e., evidence of the ‘male-taller’ norm), and (ii) individuals who scored higher on sexist attitudes, lower on feminist attitudes and/or described themselves as being traditionally feminine/masculine would have stronger height preferences, in terms of either the discrepancy between their own height and ideal partner height or the level of importance placed on partner height. Based on the free-response questions, we examined the reasons that women and men provided for why height was important to them and their views on the role of societal norms.

## Methods

### Participants

The participants (*n* = 242) were recruited via the Prolific platform (www.prolific.co) and consisted of 122 women and 120 men. The sample size was estimated using G*Power (v3.1.9; effect size = 0.30, power = 0.80 and two-tailed alpha = 0.02: see Sect. *2.3 Data collation and statistical analyses*, which provides a rationale for adjusting alpha to account for multiple comparisons). The screening process requested that participants self-reported as heterosexual and were between 18 and 29 years of age and living in the UK. At the end of the survey, participants were asked to select an age bracket to confirm that they fit the inclusion criteria, and some participants reported being slightly older, perhaps if they had joined Prolific at a younger age and not updated their profiles. The majority of the final sample were in the 18–29 years age bracket, with a small number in the 30–39 years age bracket (female participants: ‘18–29 years’ = 105 participants, ‘30–39 years’ = 17; male participants: ‘18–29 years’ = 113, ‘30–39 years’ = 7). Participants provided informed consent at the start of the survey and were compensated with a small monetary reward for their time (£1.50). The researchers followed the British Psychological Society’s Code of Human Research Ethics, and ethical approval for this study was provided by the School of Psychology & Neuroscience, University of St Andrews, UK.

### Procedures and Materials

The questionnaires were administered via Qualtrics. After confirming their gender and age bracket, all participants were asked to complete (i) a set of height-related questions, (ii) three questionnaires about endorsement of gender roles, and (iii) two open-ended questions about the importance of height in their partner preferences and their views on societal beauty standards.

#### Height-Related Questions

Participants could respond to the following height-related questions in either metres/centimetres or feet/inches (all data were later converted to metres/centimetres): (a) ‘*What is your own height?*’ (coded as ‘*own height*’), (b) ‘*When it comes to dating*,* what exact height would your romantic partner ideally be?*’ (coded as ‘*ideal height*’), (c) ‘*When it comes to dating*,* what is the height of the shortest person that you would be willing to date?*’ (coded as ‘*minimum height*’), (d) ‘*When it comes to dating*,* what is the height of the tallest person that you would be willing to date?*’ (coded as ‘*maximum height*’), and (e) ‘*Is someone’s height an important characteristic to you when you are deciding whether to go on a date with them?*’ (coded as ‘*importance of height*’), which participants answered on a 5-point Likert scale (1 = ‘*Very unimportant*’; 3 = ‘*Neither important nor unimportant*’; 5 = ‘*Very important*’). Although participants self-reported their own height, previous research indicates that self-reporting is likely to be a sufficiently accurate representation of actual height (Bowring et al., 2012; Spencer et al., 2002).

#### Endorsement of Gender Norms

All participants completed the following three questionnaires in randomised order:

*a) Ambivalent Sexism Inventory* (ASI; original version: Glick & Fiske, 1996; short-form version: Glick & Whitehead, 2010). This study used a short-form version of the ASI, consisting of 18 items that assess benevolent and hostile sexist attitudes (e.g., ‘*Women should be cherished and protected by men*’; ‘*Women are too easily offended*’; ‘*Once a woman gets a man to commit to her*,* she usually tries to put him on a tight leash*’). Participants responded to each item using a 5-point Likert-type scale (1 = *Strongly disagree*; 3 = *Neither agree nor disagree*; 5 = *Strongly agree*), with high scores indicating high endorsement of sexist attitudes (five items required reverse coding). The short-form ASI has good psychometric properties (Rollero et al., 2014; Cronbach’s a in the current study = 0.843).

*b) Liberal Feminist Attitude and Ideology Scale* (LFAIS; original version: Morgan, 1996; short version: Koyama et al., 2004). This study used a short-form version of the LFAIS, consisting of 22 items that cover attitudes towards gender roles, feminist objectives and discrimination towards women (e.g., ‘*Men and women should be able to freely make choices about their lives without being restricted by gender*’; ‘*Men still don’t take women’s ideas seriously*’; ‘*It is insulting to the husband when his wife does not take his last name*’). Participants responded to each item using a 5-point Likert-type scale (1 = *Strongly disagree*; 3 = *Neither agree nor disagree*; 5 = *Strongly agree*), with high scores indicating high endorsement of feminist attitudes (seven items required reverse coding). The short-form LFAIS has satisfactory psychometric properties (Yurtsever et al., 2021; Cronbach’s a in the current study = 0.821).

*c) Traditional Masculinity and Femininity* Scale (TMFS; Kachel et al., 2016). The TMFS probes whether participants would describe themselves as being traditionally masculine or feminine. The prompt states, ‘*Please read the following statements and select the option that best describes how you feel about yourself*’, and participants respond to six statements using a 5-point scale (1 = *Very masculine*; 2 = *Somewhat masculine*; 3 = *Neither masculine nor feminine*; 4 = *Somewhat feminine*; 5 = *Very feminine*). The six statements are: ‘*I consider myself as…*’, ‘*Ideally I would like to be…*’, and ‘*Traditionally*,* my [interests/behaviour/outer appearance] would be considered as…*’, presented as separate statements. In order to create a single scale that represented conformity to gender norms, the responses for female participants remained coded as 1 = *Very masculine* and 5 = *Very feminine*, whereas the responses for male participants were reverse coded, so that 1 = *Very feminine* and 5 = *Very masculine*, such that high scores for both women and men were indicative of high conformity to expected gender norms. The TMF was found to have good internal reliability (Cronbach’s α in the current study = 0.834).

#### Open-Ended Questions

Two open-ended questions (adapted from Yancey & Emerson, 2016), stated, (a) ‘*Why is height important*,* or unimportant*,* to you when you select someone to date?*’ and (b) ‘*Do you believe that society’s beauty standards have influenced your height preferences? If so*,* how?*’. The responses were coded using inductive thematic analyses (Braun & Clarke, 2006). In brief, all responses were read by a single coder, who generated a set of independent categories based on the content of the responses, with each response placed into one of the categories. The resulting coding scheme was then independently employed by a second researcher to test for inter-rater reliability with the full dataset (‘*Why is height important*…’: Cohen’s κ = 0.870, *p* < 0.001; ‘*Do you believe that society’s beauty standards*…’: Cohen’s κ = 0.896, *p* < 0.001), with any necessary recoding continued until complete agreement was reached. The thematic analyses resulted in the creation of a set of categories that collectively included all of the participant responses.

### Data Collation and Statistical Analyses

The height-related questions provided data on own height, ideal partner height, minimum and maximum acceptable partner height, and the importance of height to the participant. From these values, we calculated the ratio of ideal height relative to the participant’s own height, coded as *ideal height ratio*, using different equations for male and female participants (as in Salska et al., 2008), so that, for both genders, a value greater than 1 indicates a preference for the man to be taller than the woman in the partnership (‘male taller’):

*for female participants*, *ideal height ratio* = ideal height of partner/own height.

*for male participants*, *ideal height ratio* = own height/ideal height of partner.

**Fig. 1:**
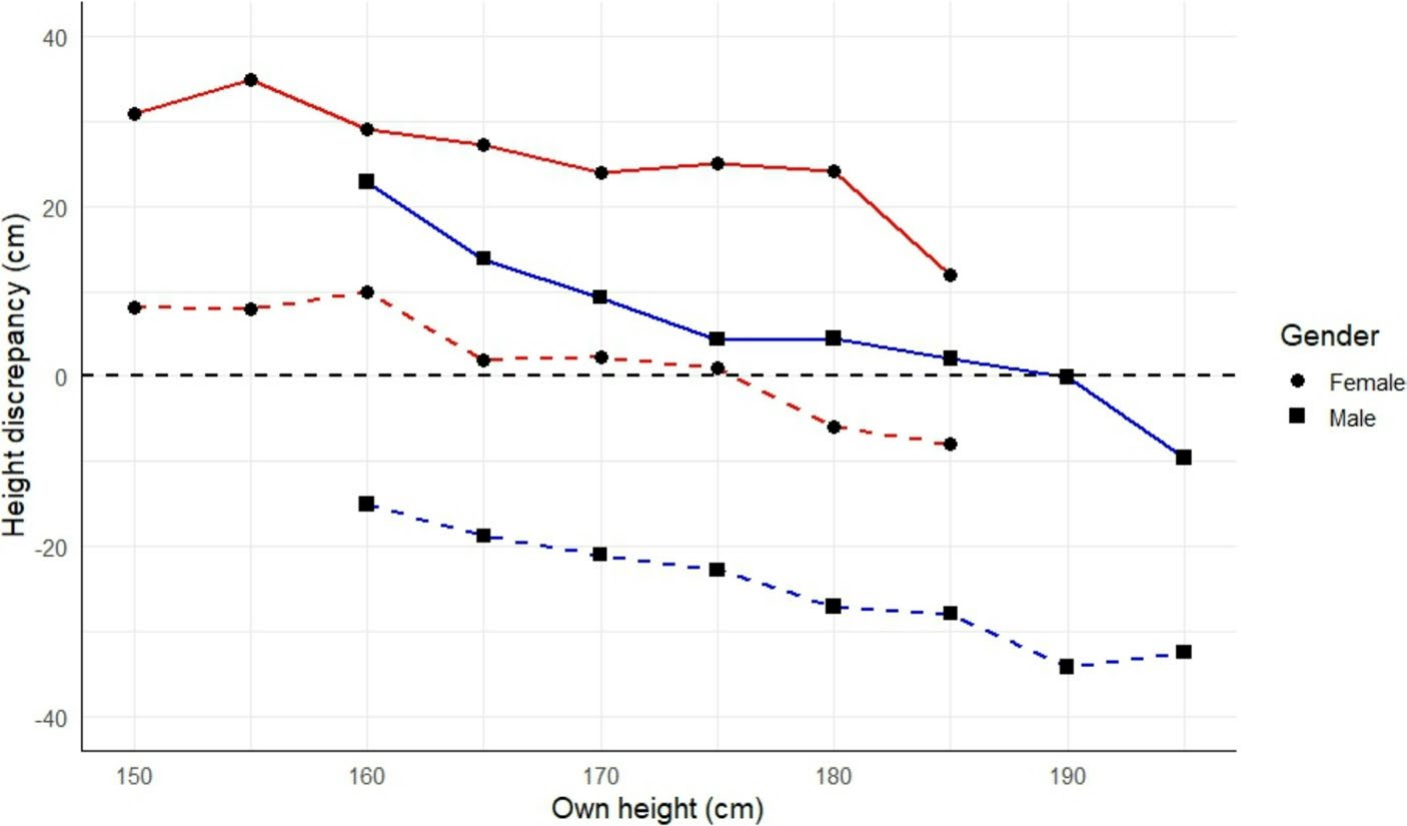
Own height (cm) plotted against discrepancy between partner’s height and own height (cm) (i.e., height discrepancy = partner height minus own height), where zero represents equal heights (positive values = partner taller than oneself; negative values = partner shorter than oneself). Solid lines = maximum acceptable height of partner; dashed lines = minimum acceptable height of partner. Circles (and red lines) = female participants (*n* = 122); squares (and blue lines) = male participants (*n* = 120)

For Fig. 1 (see Results section), we calculated the *discrepancy* (centimetres) between the shortest acceptable height and the participant’s own height (i.e., *minimum height* minus *own height*), and tallest acceptable height and the participant’s own height (i.e., *maximum height* minus *own height*) for both female and male participants; for both of these measures, a negative value indicates that the partner is shorter than oneself, and vice versa for positive values.

All statistical analyses were conducted in SPSS (v28). The assumptions of parametric statistics were checked using measures of normality (skewness and kurtosis) and equality of variance (Levene’s tests). Among male participants, one outlier was found in the *minimum height* data (where an unusually short height was entered), and one outlier was found for TMF scores; as removal of these datapoints did not affect the results, the full datasets are presented, given the relative robustness of parametric analyses to deviations from normality (Finch, 2005). A MANOVA was used to examine gender differences in responses to the height and gender role questions, and effect sizes are reported (partial eta squared, *η*^*2*^). To assess whether female and male participants differed in their ideal height preferences, one-sample t-tests were used to examine whether mean *ideal height* values were significantly different from 1 (where 1 represents same height as oneself), and effect sizes are reported (Cohen’s *d*). To assess whether participants’ responses on the gender norm questionnaires correlated with ideal height preferences and whether height is important to them, Pearson’s correlations were conducted separately for each gender to examine the relationships between height measures (*own height*, *ideal height*, *ideal height ratio*, *importance of height*,* minimum height*, *maximum height*) and gender role measures (ASI, LFAIS, TMFS). To account for multiple comparisons, the Benjamani–Hochberg method was implemented, using a false discovery rate of 0.05, which effectively produced an alpha value of 0.02 (i.e., significance level of *p* ≤ 0.02). Likelihood tests were used to analyse the coded qualitative dataset.

## Results

### Height Preferences

#### Own Height Versus Ideal Height of Partner

Female participants were approximately 12 cm shorter on average than male participants, and this gender difference in *own height* was significant (m/f ratio = 1.07; *F*_1, 240_ = 177.95, *p* ≤ 0.001, *η*^*2*^ = 0.426; Table 1). For female participants, the *ideal height* of a potential partner was, on average, considerably taller (around 16 cm) than the average female height (181.23 cm versus 165.32 cm; m/f ratio = 1.10) and 3.73 cm taller than the average height of the male participants in this study. For male participants, *ideal height* was approximately 11 cm shorter than the average male height (166.87 cm versus 177.50 cm; m/f ratio = 1.06) and slightly taller (1.55 cm) than the average height of female participants. The gender difference in *ideal height* was significant (*F*_1, 240_ = 261.74, *p* ≤ 0.001, *η*^*2*^ = 0.522; Table 1). In both genders, participant’s *own height* correlated positively with the *ideal height* of the partner (female: *r* = 0.443, *p* < 0.001; male: *r* = 0.344, *p* < 0.001; Table 2a and Table 2b respectively), which indicates, on average, taller participants ideally preferred taller partners, and shorter participants preferred shorter partners, in absolute terms.

**Table 1:** Means (± SEMs) of height-related variables, ‘importance of height’ measure (1–5 scale), and gender norm endorsement measures (1–5 scales) for female (*n* = 122) and male (*n* = 120) participants (ASI: ambivalent sexism Inventory; LFAIS: Liberal feminist attitude and ideology Scale; TMFS: traditional masculinity and femininity Scale)

	Female participants	Male participants
Own height (cm)	165.32 ± 0.62	177.50 ± 0.67***
Ideal height (cm)	181.23 ± 0.61	166.87 ± 0.64***
Ideal height ratio	1.10 ± 0.004	1.06 ± 0.005***
Importance of height	3.07 ± 0.10	2.37 ± 0.11***
Minimum height (cm)	168.96 ± 0.76	151.93 ± 1.06***
Maximum height (cm)	192.73 ± 0.76	182.12 ± 0.91***
ASI	2.41 ± 0.05	2.79 ± 0.05***
LFAIS	4.18 ± 0.04	3.66 ± 0.05***
TMFS	3.91 ± 0.05	3.92 ± 0.06

**Table 2:** Pearson’s correlation matrices for data collected from (a) women, and (b) men. *** *p* ≤ 0.001, ** *p* ≤ 0.01, * *p* ≤ 0.02

	Own height	Ideal height	Ideal height ratio	Importance	Minimum	Maximum	ASI	LFAIS	TMFS
a. Women									
Own height	-								
Ideal height	0.443***	-							
Ideal height ratio	-0.593***	0.458***	-						
Importance	0.134	0.297***	0.124	-					
Minimum	0.394***	0.594***	0.139	0.451***	-				
Maximum	0.425***	0.650***	0.162	0.035	0.317***	-			
ASI	-0.09	-0.03	0.059	0.328***	0.137	-0.179	-		
LFAIS	0.078	0.107	0.021	-0.238**	0.045	0.077	-0.594***	-	
TMFS	0.028	0.004	-0.026	0.182	0.097	-0.034	0.054	0.017	-
b. Men									
Own height	-								
Ideal height	0.344***	-							
Ideal height ratio	0.565***	-0.580***	-						
Importance	-0.061	-0.084	0.01	-					
Minimum	0.229*	0.378***	-0.148	0.206	-				
Maximum	0.367***	0.364***	0.009	-0.352***	-0.274**	-			
ASI	-0.136	-0.153	0.008	0.138	0.144	-0.187	-		
LFAIS	0.217*	0.220*	-0.002	-0.032	0.036	0.132	-0.614***	-	
TMFS	-0.036	-0.125	0.084	0.11	0.212*	-0.215*	0.276**	-0.311***	-

#### Own Height Versus Ideal Height of Partner and Ideal Height Ratio

For both female and male participants, the *ideal height ratio* was significantly different from 1 (female participants: *t*_121_ = 23.24, *p* ≤ 0.001, *d* = 2.104; male participants: *t*_119_ = 13.81, *p* ≤ 0.001, *d* = 1.260), indicating that, on average, both women and men preferred a partner who was not the same height as themselves. The *ideal height ratio* was significantly larger for women than men (*F*_1, 240_ = 26.72, *p* ≤ 0.001, *η*^*2*^ = 0.100; Table 1), confirming that, on average, women preferred a larger height discrepancy than did men. *Own height* correlated negatively with *ideal height ratio* in female participants (*r* = −0.593, *p* < 0.001; Table 2a) and positively with *ideal height ratio* in male participants (*r* = 0.565, *p* < 0.001; Table 2b), which indicates that shorter women and taller men exhibited preferences for the largest height discrepancies.

#### Importance of Height Versus Ideal Height of Partner and Ideal Height Ratio

When asked whether height is an important factor when deciding on a potential partner, almost half of female participants (43.4%) responded that height was either ‘*important*’ or ‘*very important*’ to them, compared to only around a quarter of male participants (25.8%) who selected these options. In contrast, the majority of male participants responded that height was ‘*unimportant*’ or ‘*very unimportant*’ to them (63.3%). The mean *importance of height* scores differed significantly between the genders (*F*_1, 240_ = 23.23, *p* ≤ 0.001, *η*^*2*^ = 0.088; Table 1). The *importance of height* correlated positively with *ideal height* in female participants (*r* = 0.297, *p* < 0.001; Table 2a) but not in male participants, indicating that women who placed importance on height preferred a tall partner in absolute terms. The *importance of height* did not correlate with *ideal height ratio* in either female or male participants (Table 2a and Table 2b respectively), indicating that the size of the discrepancy between own and ideal partner height was not larger in those who reported that height was important to them.

#### Minimum and Maximum Height of Partner

On average, the *minimum height* that women deemed as acceptable in a potential partner was somewhat taller (approximately 3.6 cm) than the average female height (168.96 cm versus 165.32 cm; Table 1), suggesting a general lack of willingness among women to accept a female-taller partnership, and the *maximum height* that women deemed acceptable was, on average, approximately 27 cm taller than the average female height (192.73 cm versus 165.32 cm). On average, the *minimum height* that men deemed as acceptable in a potential partner was around 25.6 cm shorter than average male (151.93 cm versus 177.50 cm), and the *maximum height* that men deemed acceptable was taller (4.62 cm) than the average male height (182.12 cm versus 177.50 cm), indicating a general willingness to consider a female-taller partnership. The gender differences in mean *minimum height* (*F*_1, 240_ = 171.12, *p* ≤ 0.001, *η*^*2*^ = 0.416) and mean *maximum height* (*F*_1, 240_ = 80.92, *p* ≤ 0.001, *η*^*2*^ = 0.252) were significant.

Consistent with assortative mating, both the *minimum* and *maximum* height acceptable in a partner correlated positively with *own height* and *ideal partner height* in women and men (*r* values in Table 2a and Table 2b respectively). Figure 1 provides a visualisation of this finding: the minimum and maximum acceptable height discrepancies varied according to the participant’s own height. The figure also demonstrates that men were more likely than women to state that they would accept a female-taller partnership, as shown by the comparison between the points at which the average *minimum height* and *maximum height* lines cross zero for female and male participants respectively. The positive correlation between *minimum* and *maximum* acceptable height in female participants (*r* = 0.317, *p* < 0.001; Table 2a), suggests that women who prefer a tall partner are also least accepting of a short partner, whereas the negative correlation between these values in male participants (*r* = −0.274, *p* = 0.002; Table 2a) suggests that some men are willing to accept a large range of absolute partner heights.

#### Minimum and Maximum Partner Height Versus Importance of Height

In female participants, the *minimum* acceptable partner height correlated positively with the *importance* placed on height (*r* = 0.451, *p* < 0.001; Table 2a), indicating that women who placed greater importance on height were less likely to find a short partner acceptable. Similarly, in male participants, the *maximum* acceptable partner height correlated negatively with the *importance* placed on height (*r* = −0.352, *p* < 0.001; Table 2b), indicating that men who placed greater importance on height were less likely to find a tall partner acceptable.

### Relationships between Endorsement of Gender Roles and Height Measures

#### Correlations between Gender Endorsement Scores

Pairwise correlations showed that ASI scores correlated negatively with LFAIS scores for both women (*r* = −0.594, *p* < 0.001; Table 2a) and men (*r* = −0.614, *p* < 0.001; Table 2b), which indicates that participants who scored high on sexism were likely to score low on feminism, and vice versa. In male participants, TMFS scores correlated positively with ASI (*r* = 0.276, *p* = 0.002; Table 2b) and negatively with LFAIS (*r* = −0.311, *p* < 0.001), indicating that male participants who described themselves as masculine scored higher on sexism and lower on feminist attitudes. In contrast, the extent to which female participants described themselves as feminine did not correlate with either sexist or feminist attitudes (*r* values in Table 2a).

#### Gender Role Endorsement Versus Own Height and Ideal Height of Partner

On average, female participants scored lower on the ASI (*F*_1, 240_ = 31.18, *p* ≤ 0.001, *η*^*2*^ = 0.115), and higher on the LFAIS (*F*_1, 240_ = 55.94, *p* ≤ 0.001, *η*^*2*^ = 0.189), than male participants (Table 1). Mean scores on the TMFS did not differ between women and men (*F*_1, 240_ = 0.02, n.s.; Table 1), which indicates that, on average, female and male participants described themselves to an equal extent as conforming to gender norms (i.e., femininity and masculinity respectively). In female participants, neither *own height* nor *ideal height* of a partner correlated with AIS, LFAIS or TMFS (*r* values in Table 2a). In male participants, *own height* and *ideal height* of a partner did not correlate with AIS or TMFS (*r* values in Table 2b), but both measures correlated positively with LFAIS (*own height*: *r* = 0.217, *p* = 0.017; *ideal height*: *r* = 0.220, *p* = 0.016), meaning that men who endorsed feminist attitudes were more likely to be taller, and prefer a tall partner in absolute terms, than men who scored lower on feminist attitudes.

#### Gender Role Endorsement Versus Ideal Height Ratio and Importance of Height

The *ideal height ratio* did not correlate with any of the gender role measures (i.e., ASI, LFAIS, TMFS) in either female or male participants (Table 2a and Table 2b respectively). However, in female participants, the *importance of height* correlated positively with ASI (*r* = 0.328, *p* < 0.001) and negatively with LFAIS (*r* = −0.238, *p* = 0.008; Table 2a), indicating that female participants who placed greater importance on height were more likely to score high on sexist attitudes and low on feminist attitudes. In contrast, the *importance of height* did not correlate with any of the gender role scores in male participants (Table 2b).

#### Gender Role Endorsement Versus Minimum and Maximum Height of Partner

In female participants, the *maximum* and *minimum height* acceptable in a partner did not correlate significantly with any of the gender role endorsement measures (*r* values in Table 2a). In men, the extent to which participants described themselves as traditionally masculine (TMFS) correlated positive with *minimum height* (*r* = 0.212, *p* = 0.020; Table 2b) and negatively with *maximum height* (*r* = −0.215, *p* = 0.019), meaning that male participants who described themselves as masculine were willing to accept a narrower range of partner heights compared to men who described themselves as conforming less to traditional masculine gender norms.

### Participant-Generated Responses

#### Explanations for Importance of Height

As noted above, almost half of female participants (43.4%) responded that height was either ‘*important*’ or ‘*very important*’, whereas only a quarter of male participants (25.8%) rated height as either of these categories. For these participants who stated that height was *important*/*very important*, qualitative analyses of the free-text responses to the question ‘*why is height important*’ generated five categories of responses: (i) *physical attraction*, (ii) *aesthetics*, (iii) *femininity/protection*, (iv) *masculinity/dominance/maturity* and (v) *practicalities* (see example statements in Table 3). The percentage of responses that fell into these coding categories differed significantly between female and male participants (likelihood ratio = 9.967, *p* = 0.041; Table 4). For female participants, most of the responses related to physical attraction, aesthetics, and feelings of femininity/protection, whereas most of the responses from male participants related to physical attraction, aesthetics, and feelings of masculinity/dominance/maturity.

**Table 3:** Categories that were generated from the participants’ open-ended responses to the question of ‘why is height important to you when you select someone to date’, plus example statements from each of the coding categories (F = female participant; M = male participant)

Why is height important?	Example statements
Physical attraction	‘I find taller men attractive’ (F); ‘I prefer a partner that’s a bit shorter than me because I find it more attractive’ (M)
Aesthetics	‘I don’t want to look odd’ (F); ‘Because I am not tall myself, I wouldn’t want somebody much taller than me, as it would make me feel uncomfortable’ (M)
Femininity/protection	‘I prefer to date someone taller as I would feel protected by them’ (F); ‘I like to feel small in comparison to my partner – it makes me feel feminine’ (F)
Masculinity/dominance/maturity	‘It seems to feel ‘right’ and makes them seem the more dominant in the relationship’ (F); ‘Being significantly shorter than your partner can make you feel less masculine’ (M)
Practicality	‘I think it would be easier if my partner was the same height or taller than me more for instances like having a conversation’ (F); ‘I am tall so I would prefer someone who is also tall, it is also better for my back’ (M)

#### Role of Societal Beauty Standards

In response to the question of whether participants believed that society’s beauty standards had influenced their height preferences, female participants who reported that height was important/very important to them were equally likely to answer ‘yes’ (44.0%) and ‘no’ (42.0%), whereas male participants who stated that height was important/very important to them were most likely to answer ‘no’ (54.8%, versus 35.5% ‘yes’) (Table 4). Many respondents gave a one-word answer to this question or did not explain what aspects of societal standards influence their preference. However, some participants did mention that either social norms influenced their height preferences (N = 7 F, 8 M; for example, female participant: ‘Yes, most definitely – I feel it is a social norm for men to be taller in a relationship’; male participant: ‘Definitely, it is more socially acceptable for the girl to be shorter’), or media portrayals influenced their height preferences (N = 7 F, 3 M; for example, female participant: ‘Yes, I do think so, as this what is portrayed in social media, and they are always mentioning tall, dark and handsome’; male participant: ‘Yes, social media brainwashed society’).

**Table 4:** Percentage of response from female participants and male participants to the two open-ended questions that were coded under each of the coding categories, plus the total number of responses received for each question

		% of female responses	% of male responses
Why is height important?	Physical attraction	25.7	28.6
	Aesthetics	25.7	28.6
	Femininity/protection	31.4	0
	Masculinity/dominance/maturity	11.4	28.6
	Practicality	5.7	14
	Number of responses (n)	35	14
Has society's beauty standards influenced your height preferences?	Yes	44.0	35.5
	No	42.0	54.8
	Maybe/partially	14.0	9.7
	Number of responses (n)	50	31

## Discussion

On average, women in this study reported a preference for a partner taller than themselves, and men reported a preference for a partner shorter than themselves, which is consistent with the male-taller norm, and height was rated as more important by women than men. Female participants appeared to be less willing to compromise in their partner choice; for instance, the average minimum height that women deemed acceptable was still taller than the average female height in this sample. The ideal height ratio was larger for women than men, suggesting a potential mismatch between the genders in terms of their absolute preferences. Contrary to our predictions, ideal height ratio did not correlate with any of the gender role endorsement measures in either women or men. However, women who placed greater importance on height scored higher on sexism, lower on feminism and were less likely to find a short partner acceptable. Men who placed greater importance on height, and men who described themselves as more traditionally masculine, were less willing to accept a tall partner. Among participants who rated height as important, women wanted to feel feminine or protected, while men wanted to feel masculine, dominant or mature relative to their partner. Collectively, these findings indicate that some aspects of height preferences are related to an individual’s endorsement of, and alignment with, societal gender norms in this sample. Assuming that these preferences translate into real-life partner choices, these results suggest that cultural factors could influence selection acting on height across populations and time periods.

In line with previous studies demonstrating a male-taller norm in Western populations (e.g., Salska et al., 2008; Shepperd & Strathman, 1989; Swami et al., 2008), women stated a preference for a partner taller than themselves, and men stated a preference for a partner shorter than themselves. On average, ideal partner height for female participants was almost 4 cm taller than the average male height in this sample, and the ideal partner height for male participants was around 1.6 cm taller (not shorter) than the average female height. These absolute values are very similar to those reported in previous studies of Western samples (e.g., 4 cm and 1.1 cm respectively: Swami et al., 2008; also Salska et al., 2008). Our findings are therefore consistent with previous evidence that women seek a larger discrepancy between their own height and their partner’s height than do men (e.g., Stulp et al., 2013b). The ideal height ratio in our study was larger on average for women (1.10) than men (1.06), and these ratios are similar to those reported in previous studies of Western samples (e.g., 1.10 and 1.05 respectively: Salska et al., 2008; also see Swami et al., 2008). These findings are potentially consistent with the idea of directional sexual selection for tallness in men in these samples.

Participant’s own height correlated with ideal height ratio, indicating that shorter women and taller men preferred larger discrepancies between their own height and their partner’s height, which could partly reflect the realistic expectations faced by individuals at the extremes of the height distributions. As reported by Swami and colleagues (2008), shorter women preferred shorter men in absolute terms, and taller men preferred taller partners while also preferring a larger height difference. Women in this study placed more importance on height than men (as in Yancey & Emerson, 2016), and the minimal height that women deemed acceptable in a partner was, on average, taller than the average female height in this sample (as in Salska et al., 2008). These findings suggest that women, except for very tall women, are generally unwilling to consider a female-taller partnership. In contrast, the maximum height deemed acceptable by men was taller than the average male height and, across a broad range of heights, men were willing to accept a partner taller than themselves. More specifically, in our UK-based sample, only very tall men (≥ 190 cm) were likely to find a partner taller than themselves unacceptable (similar to a European sample: Stulp et al., 2013b). In comparison, in a US-based sample (Salska et al., 2008), men of average height and above (≥ 174 cm) were generally unwilling to accept a taller partner, which suggests that cultural factors, such as definitions of masculinity, might influence willingness to contravene male-taller norms, or that these norms have changed since publication of this previous study.

On average, women scored lower on sexist attitudes, and higher on feminist attitudes, than men, consistent with previous studies showing that sexist and feminist views exhibit gender differences, and are negatively related to each other, in Western samples (e.g., Wareham et al., 2024). Female and male participants, respectively, described themselves as feminine or masculine to a similar extent. None of these measures of gender norm endorsement correlated with own height, ideal height or ideal height ratio, except that men who endorsed feminist attitudes were more likely to be taller, and prefer a taller partner in absolute terms, than men who scored low on feminism. However, the importance placed on height did relate to these gender norm measures. More specifically, women who placed a greater importance on height scored higher on sexist attitudes, scored lower on feminist attitudes and were less willing to find a short partner acceptable than women who placed less importance on height. These women might not prefer taller men than other women do in absolute terms, but they might perhaps be more likely to prioritise height when making real-life decisions about potential partners. Men who placed greater importance on height, and men who described themselves as more traditionally masculine, were less willing to accept a tall partner than men who scored lower on these measures. Men who described themselves as more traditionally masculine were also less willing to accept a short partner. Our findings did not exactly replicate previous studies that found a relationship between ideal height ratio and sexist attitudes in large UK-based samples (Swami et al., 2008, 2010); the relatively small effect sizes for some of these previous findings, and our more stringent alpha value, might have reduced the likelihood of replicating those potential associations. Our results suggest that gender role attitudes might relate more strongly to the *importance* placed on height during partner choice, rather than to preferences for specific height ratios per se.

When participants were asked why height was important to them, the responses from female participants related to feeling feminine and protected, while male participants mentioned wanting to feeling masculine, dominant and mature relative to their partners. Both genders also mentioned the importance of physical attraction and aesthetics. Women were more likely than men to agree that societal beauty standards had influenced their height preferences. Many Western societal beauty standards, including the male-taller norm, emphasise power differentials between the genders and promote the notion that women must be submissive and that men must be dominant (Bareket & Fiske, 2023). Such beauty standards are potential vehicles for perpetuating gender inequalities. Yancey and Emerson (2016) similarly reported that female participants in a US-based sample mentioned protection and security when asked about their height preferences, while both genders mentioned feeling socially awkward about contravening the male-taller norm. A study of Filipino youth reported that participants cited protection, power and social acceptability as reasons why height is important to them in romantic relationships (Taduran, 2021). The perpetuation of stereotyped expectations about height differences in romantic relationships could negatively impact an individual’s satisfaction with their own height and their partner’s height (Stulp et al., 2013b), thus impacting wellbeing or relationship success.

In terms of evolutionary processes influencing human height dimorphism, the strength of selection acting on height might vary across populations depending upon whether members of those populations conform to particular social norms related to gender roles. In our study, women stated a preference for partners that were taller than the average male height in our study population, which means that selection for male tallness could potentially occur in this population, if such directional effects were consistently shown over a sufficiently long timescale. However, as argued previously (Gahtan & Mark, 2013; Stulp & Barrett, 2016), it is unclear whether social norms related to partner preferences are sufficiently stable or strong to influence height dimorphism. While the average height of a population can vary across relatively small geographical areas and time periods (Cox et al., 2023; Stulp et al., 2023), this variability is most likely to reflect environmental and epigenetic factors that influence height, such as nutritional status and stress exposure (Perkins et al., 2016; Simeone & Alberti, 2014). For instance, the rapid increase in average height over the past century in some European countries is not thought to have resulted from differential reproductive success based on height (Gahtan & Mark, 2013; Stulp et al., 2023). Therefore, while cultural evolutionary process could influence height dimorphism, and could impact selection acting on genetic variants that influence height, whether such processes have acted within any human populations remains an open question.

In this study, both women and men exhibited a preference for male-taller partnerships, and women appeared to be less willing than men to contravene this norm. Women who placed importance on the height of their partner endorsed more traditional gender norms and were less likely to accept a short partner, and men who described themselves as more traditionally masculine were less accepting of a taller partner. The evidence that gender norms are related to the importance placed on height suggests that such preferences are flexible, variable and responsive to prevailing social norms that are likely to vary across populations and time periods. This conclusion is consistent with the evidence that the male-taller norm is not found across all contemporary populations (e.g., Sorokowski & Butovskaya, 2012; Sorokowski et al., 2012, 2020). While actual partner choice decisions were not examined in our study, previous research has suggested that self-reported preferences in experimental settings are good indicators of actual partner choice (e.g., Conroy-Beam & Buss, 2016; Dreibe et al., 2023), and female-taller partnerships in real-life datasets are less common than expected by chance in Western populations (e.g., Gillis & Avis, 1980; Sear et al., 2004; Stulp et al., 2013b). Thus, culturally influenced partner preferences could potentially impact height dimorphism in both contemporary populations and past selective environments. The relative influence of social norms, flexible mate preferences and environmental factors on human height are yet to be fully disentangled.

## Data Availability

The research data underpinning this publication are available open access at: 10.17630/34becbed-bb82-4743-8eeb-08e987c59a81.
